# Thermographic assessment of surface temperature distribution and agreement between a thermographic camera, infrared thermometer, and rectal temperature in exercising horses under tropical climate conditions

**DOI:** 10.14202/vetworld.2026.3899-3914

**Published:** 2026-09-07

**Authors:** Jakkawat Pongsumpun, Ashannut Isawirodom, Phawita Sangsasithorn, Kanokpich Wong-aree, Nuttapon Satumay, Panuwat Yamsakul, Wanpitak Pongkan, Porrakote Rungsri, Kannika Na Lampang

**Affiliations:** 1 Faculty of Veterinary Medicine, Chiang Mai University, Chiang Mai 50100, Thailand.; 2 Royal Stable Unit Equine Medical and Rehabilitation Service Centre, Bangkok 10400, Thailand.; 3 Department of Veterinary Medicine, Faculty of Veterinary Medicine, Chiang Mai University, Chiang Mai 50100, Thailand.; 4 Research Center for Veterinary Biosciences and Veterinary Public Health, Faculty of Veterinary Medicine, Chiang Mai University, Chiang Mai 50100, Thailand.

**Keywords:** ambient temperature, equine thermography, exercise physiology, heat stress, infrared thermometer, surface temperature, thermographic camera, tropical climate

## Abstract

**Background and Aim:**

Infrared thermography has become an increasingly valuable non-invasive imaging technique for monitoring physiological status and detecting abnormalities in horses. However, its application under tropical environmental conditions remains insufficiently investigated, particularly regarding the influence of ambient conditions on measurement accuracy and agreement with conventional temperature assessment methods. This study aimed to evaluate the agreement among a thermographic camera (TC), infrared thermometer (IR), and rectal temperature (RT), characterize body surface temperature distribution before and after exercise, and determine the effects of environmental conditions on temperature measurements in exercising horses under tropical climate conditions.

**Materials and Methods:**

Thirty-one clinically healthy horses performing schooling, training, or lunging exercises were enrolled in this prospective repeated-measures observational study. Temperature measurements were obtained using RT, IR at the medial canthus, and TC across the eye, neck, shoulder, back, trunk, and hindquarters regions before exercise, immediately before and after exercise, and during recovery. Environmental variables, including ambient temperature and relative humidity, were simultaneously recorded. Temperature differences among anatomical regions, exercise stages, and measurement methods were analyzed using linear mixed-effects models. Agreement between methods was assessed using Bland-Altman analysis, whereas environmental influences were evaluated using Kendall’s rank correlation.

**Results:**

No significant differences were observed between the left and right body sides or among exercise activities. RT consistently recorded the highest temperatures, followed by IR and TC. Thermographic assessment demonstrated that the eye exhibited the highest surface temperature under resting conditions, whereas the back, trunk, and hindquarters showed the greatest post-exercise temperature increases (p < 0.001). Outdoor measurements produced significantly higher TC temperatures than indoor assessments (p < 0.001). IR demonstrated closer agreement with RT, with a mean difference of 1.02°C, whereas TC underestimated RT by 2.88°C and exhibited wider limits of agreement. Ambient temperature showed a strong positive correlation with TC measurements (r = 0.52, p < 0.001) but had minimal influence on IR measurements, indicating superior environmental stability of IR. Surface temperatures generally returned to baseline during the recovery period.

**Conclusion:**

Thermographic imaging provides comprehensive visualization of regional surface heat distribution and effectively identifies exercise-induced thermal changes in horses under tropical conditions. However, its measurements are substantially influenced by environmental temperature and should not be used as a surrogate for core body temperature. In contrast, IR demonstrated greater agreement with RT and superior resistance to environmental variation, making it a practical alternative for rapid estimation of body temperature when rectal measurement is impractical. These findings provide valuable guidance for selecting appropriate temperature monitoring techniques for equine health assessment, exercise monitoring, and welfare management in tropical climates.

## INTRODUCTION

Body temperature is one of the most important physiological parameters, and maintaining it within a narrow, well-defined range is essential for normal metabolic and cellular function [1, 2]. In healthy adult horses, normal RT ranges from 37.2°C to 38.3°C [3]. This physiological baseline can be altered by both pathological and non-pathological factors. Pathological conditions, including bacterial, viral, and parasitic infections, inflammation, and toxin exposure, induce fever through hypothalamic regulation of thermoregulatory responses [4], whereas shock and severe blood loss may result in hypothermia. Conversely, non-pathological factors, particularly exercise and stress, increase body temperature through normal physiological mechanisms rather than disease processes. During exercise, approximately 70%–80% of the energy generated by contracting skeletal muscles is dissipated as heat rather than converted into mechanical work, and heat production increases rapidly with exercise intensity, resulting in elevated circulating blood and core body temperatures [5]. Similarly, stress activates the hypothalamic-pituitary-adrenal (HPA) axis, leading to cortisol release and thermoregulatory responses that contribute to transient hyperthermia [6, 7]. Therefore, body temperature is not only a fundamental indicator of physiological status but also an essential clinical parameter for disease diagnosis, treatment planning, and therapeutic monitoring [8].

Several methods are available for measuring body temperature in horses, ranging from conventional contact thermometers to non-contact infrared technologies. RT remains the clinical reference standard because it most accurately reflects core body temperature. However, rectal temperature (RT) measurement requires restraining the horse for up to 60 seconds to obtain a reading, thereby increasing stress and posing potential safety risks to both the horse and handler. Infrared thermometers (IR) and infrared thermography (IRT) provide rapid, non-contact alternatives by detecting infrared radiation emitted from the skin or mucosal surfaces [9–11]. These technologies substantially reduce handling time while minimizing stress associated with physical restraint. Unlike IR, which measures temperature at a single anatomical point, IRT converts infrared radiation into thermal images, enabling visualization and quantitative analysis of body surface temperature distribution [11]. Consequently, IRT has become an increasingly valuable imaging modality in veterinary medicine for detecting localized inflammation, monitoring tissue healing, assessing physiological responses, and complementing conventional diagnostic imaging techniques [12–19]. In addition, IRT has been used to evaluate stress responses and animal welfare, particularly in athletic horses undergoing training and competition [20–22].

Despite these advantages, both IR and IRT have important limitations that should be considered during clinical interpretation. Temperature measurements obtained using these techniques are highly dependent on the anatomical site being evaluated. Previous studies have demonstrated that the medial canthus of the eye provides the closest approximation to core body temperature because of its minimal hair coverage and abundant vascular supply, allowing rapid reflection of changes in blood flow and thermoregulation [22–24]. In contrast, the shoulder, back, and thigh regions generally exhibit higher surface temperatures because of their greater muscle mass and metabolic activity [12, 25], whereas the distal thoracic and pelvic limbs have the lowest temperatures because of their predominance of tendinous tissue and relatively limited blood perfusion [14]. Elevated surface temperatures, however, are not disease-specific and may arise from numerous physiological and environmental factors, thereby limiting diagnostic specificity. Furthermore, both IR and IRT measure only surface temperatures and provide limited information regarding deeper tissues. Although IRT offers comprehensive visualization of regional thermal distribution, variability in equipment quality, image acquisition protocols, and operator expertise further supports its use as an adjunctive rather than a standalone diagnostic modality in equine practice [22, 26].

Another important limitation of IRT is its susceptibility to environmental conditions. Ambient temperature, solar radiation, and air movement can substantially influence thermal images and reduce measurement accuracy [22, 26–28]. Although these environmental effects have been recognized, they have not been comprehensively investigated, particularly under persistently high ambient temperatures and humidity characteristic of tropical climates [22, 26–28]. Consequently, the reliability of IRT for monitoring exercising horses under tropical environmental conditions remains insufficiently understood.

Although IRT has been increasingly incorporated into equine veterinary medicine, most published studies have been conducted under temperate climatic conditions, where environmental influences on thermal measurements are comparatively limited. Consequently, there is limited evidence regarding the performance, accuracy, and agreement of TC, IR, and RT measurements under tropical environmental conditions characterized by high ambient temperatures and humidity. Furthermore, few studies have simultaneously evaluated regional body surface heat distribution during exercise and recovery while assessing the effects of environmental variables on different temperature measurement techniques. The magnitude of environmental interference and its impact on the clinical interpretation of thermal images therefore remain poorly defined, limiting the practical application of these technologies for monitoring exercising horses and detecting heat-related abnormalities in tropical regions.

This study aimed to evaluate the agreement among a thermographic camera (TC), IR, and RT measurements in clinically healthy horses exercising under tropical climatic conditions. In addition, the study characterized regional body surface temperature distribution before exercise, immediately after exercise, and during the recovery period using thermal imaging while investigating the influence of environmental factors on the performance of each temperature assessment method. The findings are expected to provide evidence-based guidance for selecting appropriate non-contact temperature monitoring techniques for equine clinical evaluation, exercise monitoring, and welfare assessment under tropical environmental conditions. Throughout the study, horse safety and welfare were prioritized by implementing continuous clinical monitoring and appropriate management practices to minimize the risk of stress or injury.

## MATERIALS AND METHODS

### Ethical approval

The study protocol was reviewed and approved by the Laboratory Animal Center, Chiang Mai University, Chiang Mai, Thailand (Approval No. AG001/2567, Date 21/08/2024). Written informed consent was obtained from the owners before the enrollment and participation of their horses in the study. All animal handling, clinical examinations, exercise procedures, and temperature measurements were performed in accordance with the institutional guidelines for the ethical use, care, and welfare of animals used in research.

Only clinically healthy horses were enrolled after physical examination and cardiac auscultation. Horses with pyrexia (RT >38.5°C), lameness, recent medication administration, or unwillingness to cooperate during handling were excluded to minimize avoidable welfare risks. Of the 32 horses initially screened, one was excluded because pyrexia was detected during the initial examination, resulting in 31 horses being included in the study.

The experimental procedures were designed to minimize pain, distress, and unnecessary restraint. Non-invasive temperature assessment techniques were used whenever possible, and all measurements were performed by trained personnel. The horses continued their routine low-to-moderate-intensity exercise programs rather than being subjected to additional experimental exercise. RT was deliberately not measured immediately before or after exercise because of the potential safety risks associated with obtaining rectal measurements in recently exercised horses. Throughout the experimental procedures, the horses were continuously monitored for changes in mental status and respiratory pattern and for clinical signs of hyperthermia or distress, including excessive sweating and drooling. Any procedure that could compromise animal safety or welfare was avoided. The study was conducted under practical riding-club conditions without introducing invasive experimental interventions, and each horse participated in only one study session. Appropriate acclimatization, handling, environmental protection, and recovery procedures were incorporated into the study protocol to reduce stress and environmental interference. In addition, the study was reported in accordance with the ARRIVE 2.0 guidelines [29], with the completed ARRIVE checklist provided as supplementary material.

### Study period and location

The study was conducted in April 2025 at a riding club in Bangkok, Thailand (13.773762° N, 100.545314° E). Temperature measurements were performed under typical tropical environmental conditions during routine horse management and exercise sessions.

### Animals

Clinically healthy horses with no history of lameness or systemic disease during the preceding 2 weeks were eligible for inclusion. Health status was confirmed through physical examination and cardiac auscultation before enrollment. Horses presenting with pyrexia (RT >38.5°C), lameness, administration of any medication within the previous 14 days, or unwillingness to cooperate during handling were excluded from the study.

Initially, 32 horses were screened; however, one horse was excluded because pyrexia was detected during the initial physical examination. Consequently, 31 horses (n = 31) were included in the final analysis, comprising 26 geldings and five mares. The horses ranged in age from 4 to 18 years (11.32 ± 3.57 years) and weighed between 396 and 644 kg (515.19 ± 61.03 kg). Body condition score (BCS) ranged from 3 to 5 (5-point scale) and was assessed according to Carroll and Huntington [30]. The study population included 17 mixed-breed horses, five Thoroughbreds, and nine Warmbloods. All horses had short hair coats, consisting of 27 bay, two gray, and two black horses.

The horses were members of the riding club and participated regularly in routine exercise programs. Exercise activities comprised schooling (n = 15), training (n = 11), and lunging (n = 5). During schooling and training sessions, each horse was ridden by a single rider using a saddle and bridle, whereas lunging was performed without a rider. All activities were considered low-to-moderate intensity and were conducted in an outdoor riding arena as part of the horses' normal exercise routine. Details of the exercise duration for each activity are presented in Table 1.

Sample size was determined by the availability of suitable horses during the study period. Horses were assigned to individual study sessions by independent personnel who were not involved in the study protocol, whereas the order of measurements depended on the horses' arrival times at the study site.

**Table 1:** Exercise characteristics of the study horses according to activity type.

**Activity type**	**Total (min)**	**Walk (min)**	**Trot (min)**	**Canter (min)**	**Stand (min)**
Lunging	18.2 (11–27)	3.8 (0–7)	3.8 (1–6)	10.0 (6–17)	0.6 (0–3)
Schooling	47.93 (37–57)	21.07 (10–33)	18.33 (12–37)	6.67 (0–14)	1.87 (0–7)
Training	23.64 (18–30)	8.55 (4–17)	9.82 (2–14)	4.0 (0–9)	1.73 (0–4)

Data are presented as mean (minimum–maximum).

### Study design

This prospective repeated-measures within-subject observational study was designed so that each horse served as its own control. Temperature measurements were performed at four predefined time points: 1 h before exercise (pre-examination room), immediately before exercise (pre-exercise), immediately after exercise (post-exercise), and 1 h after exercise (post-examination room). The pre- and post-examination room assessments were conducted indoors, whereas the pre- and post-exercise assessments were performed outdoors.

Each horse completed the entire experimental protocol consecutively while following its routine exercise and cooldown schedule to closely simulate practical field conditions. Measurements were performed in the following sequence: physical examination, RT measurement, IR measurement, and thermographic imaging. All temperature measurements were completed within 5 min of one another to minimize temporal variation.

Before indoor measurements, horses were not exercised, groomed, or clipped for at least 1 h. Saddles were removed before thermographic imaging, and each horse was allowed to rest for at least 10 min before entering the examination room, followed by a further 10-min acclimatization period. The examination room was physically separated from the stable, enclosed, protected from direct sunlight, and illuminated only by ceiling-mounted LED lighting to provide a calm and environmentally controlled setting.

Outdoor measurements were obtained immediately before and immediately after the exercise session in a shaded area adjacent to the riding arena to minimize artifacts caused by direct solar radiation. During these measurements, horses remained equipped with a saddle and bridle to maintain normal exercise conditions.

RT was not measured immediately before or after exercise because of safety concerns associated with obtaining rectal measurements in recently exercised horses. Throughout the study, all horses were continuously monitored for mental status, respiratory pattern, and clinical signs of hyperthermia, including excessive sweating and drooling, to ensure animal welfare.

Two study sessions were conducted daily. The morning session was performed between 6:00 and 7:00 a.m., whereas the afternoon session was conducted between 3:00 and 4:00 p.m. Each horse participated in only one study session. The overall study workflow, including animal enrollment, inclusion and exclusion criteria, and experimental procedures, is illustrated in Figure 1.

### Temperature measurement procedures

**RT:** RT was measured using a digital thermometer (Omron MC-245; Omron Healthcare, Kyoto, Japan). The thermometer was inserted 10–15 cm into the rectum, depending on the size of the horse, and maintained in position for 1 min to obtain the temperature reading.

**IR:** An IR (Philips BBL50284AE/93; Philips, Amsterdam, the Netherlands) was used to measure the temperature at the medial canthus of the eye. The probe was positioned perpendicular to the medial canthus at a constant distance of 0.5 cm. Three consecutive measurements were obtained for each horse, and the average value was used for subsequent analyses.

**TC:** A TC (FLIR C5; FLIR Systems, Wilsonville, OR, USA) was used to capture thermal images of both lateral aspects of each horse. One image of the head and one image of the body were obtained from each side, resulting in a total of 12 thermal images per horse at each sampling time. During image acquisition, horses stood squarely with the head maintained in a normal resting position while being gently restrained with a halter by a handler positioned in front of the horse.

**Figure 1:**
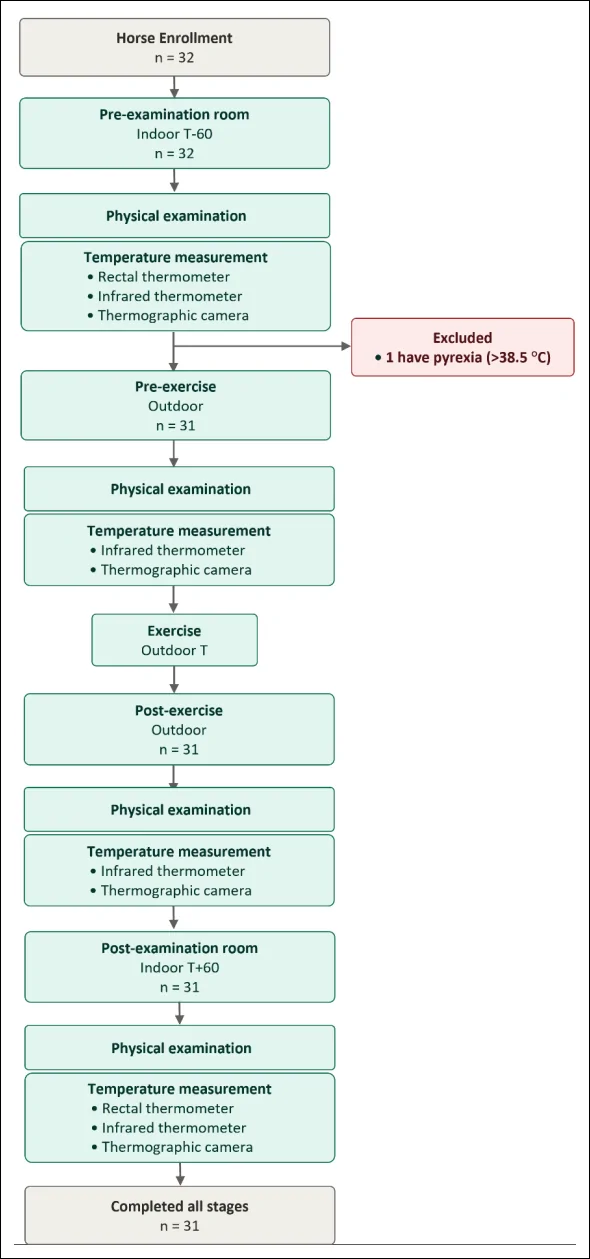
Experimental workflow illustrating animal enrollment, inclusion and exclusion criteria, and study procedures. Temperature measurements were performed at four predefined time points relative to exercise: T−60 = 1 h before exercise (pre-examination room), pre-exercise, post-exercise, and T+60 = 1 h after exercise (post-examination room).

The imaging distance was standardized using floor markers positioned 2.0 m from the horse for body imaging and 0.5 m for head imaging (Figure 2). The TC was maintained perpendicular to the horse using the integrated spirit level to ensure consistent image orientation. Before image acquisition, background temperature was measured using black non-reflective tape positioned on the same plane as the horse. Reflected temperature was determined using crumpled and flattened aluminum foil with emissivity temporarily set to 1.0. Background temperature, reflected temperature, imaging distance, emissivity, and ambient humidity recorded by the environmental monitoring station were entered into the TC before image acquisition. Automatic temperature correction was enabled, and emissivity was set to 0.98 according to previous equine thermography studies [14, 16] and published data regarding the emissivity of pigmented human skin [31].

**Figure 2:**
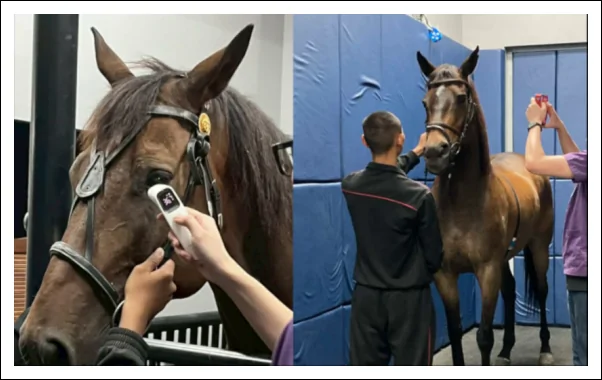
IR positioned to measure temperature at the medial canthus of the eye (left) and TC used to capture thermal images of the horse's head (right). IR = Infrared thermometer. TC = Thermographic camera.

Thermal images were exported and analyzed using FLIR Thermal Studio version 2.0.53 (FLIR Systems). Images exhibiting motion blur, poor focus, loss of anatomical border definition, or imaging artifacts were excluded from the analysis. Thermal images were processed using the Rainbow High Contrast color palette with Multi-Spectral Dynamic Imaging (MSX). Image analysis parameters, including background temperature, reflected temperature, imaging distance, emissivity, and humidity, were automatically imported from the camera settings recorded during image acquisition.

For head images, temperature at the medial canthus was measured using the software spot-meter function. Body thermal images were divided into five anatomically defined regions comprising the neck, shoulder, back, trunk, and hindquarters (Figure 3).

**Figure 3:**
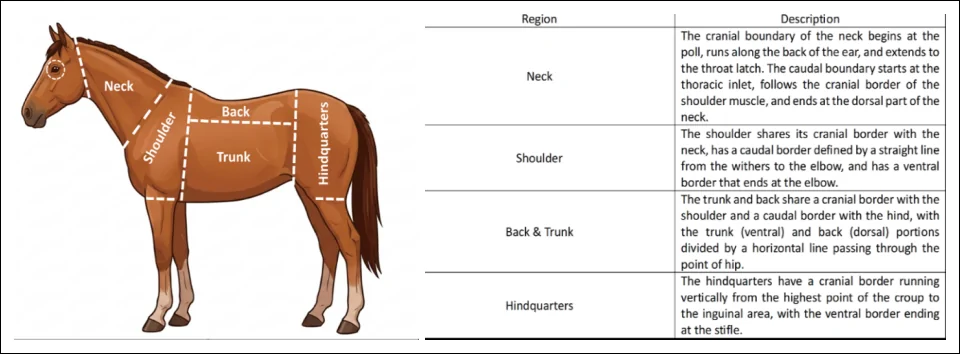
Anatomical regions used for TC temperature measurements, including the eye, neck, shoulder, back, trunk, and hindquarters. TC = Thermographic camera.

Polygonal regions of interest were manually outlined using the built-in software tools according to predefined anatomical landmarks. For outdoor images, saddle-covered areas were excluded from the analysis. The maximum temperature within each region of interest was recorded (Figures 4 and 5). Maximum temperature was selected because it represents the thermal "hot spot," which is widely used in equine thermography studies [11, 13] and is more practical for field-based monitoring. Three repeated measurements were obtained for each anatomical region, and their mean value was used for statistical analysis.

Image analysis was performed by a single evaluator who was familiar with horse identification and the study protocol to ensure consistent image interpretation. Intra-observer reliability was assessed using the two-way mixed-effects Intraclass Correlation Coefficient (ICC [3, k]). Excellent repeatability was observed, with ICC values of 1.00 for the eye, 0.99 for the trunk, and 0.98 for the neck, shoulder, back, and hindquarters.

**Figure 4:**
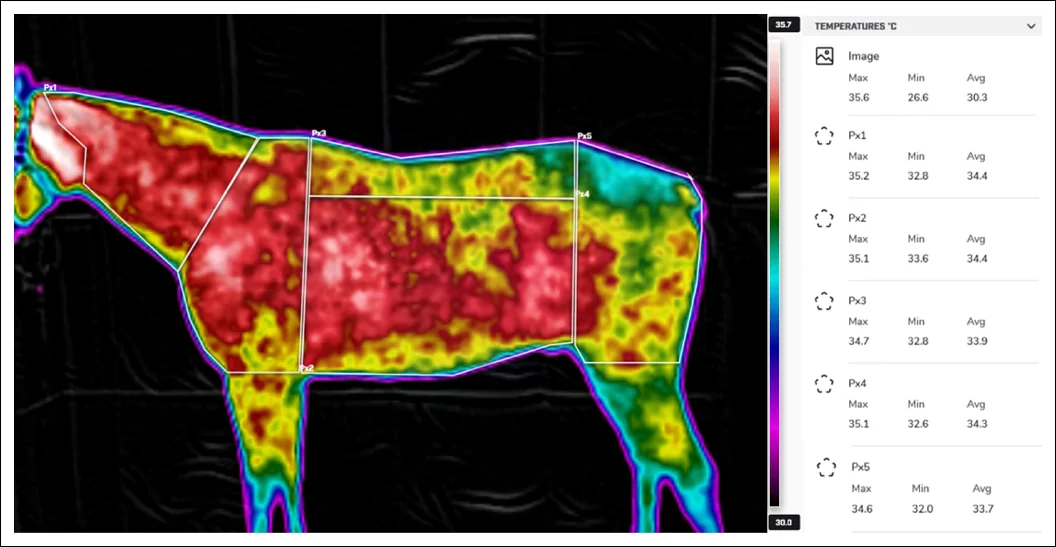
Representative thermal image illustrating the anatomical regions of interest used for analysis: Px1 = head, Px2 = shoulder, Px3 = back, Px4 = trunk, and Px5 = hindquarters, together with the corresponding maximum, minimum, and mean temperatures. Px = Polygonal region of interest.

**Figure 5:**
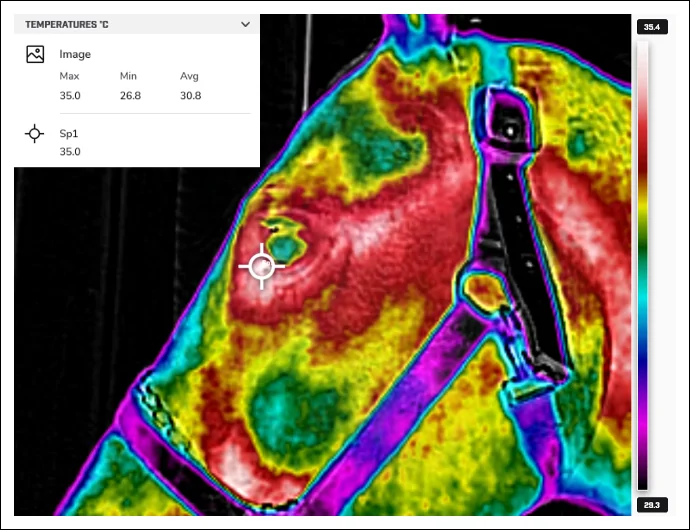
Representative thermal image of the horse's head showing the spot-meter location used to measure temperature at the medial canthus.

### Environmental monitoring

Environmental conditions were recorded using an environmental monitoring station (Nicety Meter 0371-020T; Starmeter Instruments, Shenzhen, China). Ambient temperature (°C) and relative humidity (%) were measured during both indoor and outdoor sessions. During indoor measurements, the monitoring station was positioned 2 m in front of the horse to represent the environmental conditions surrounding the animal.

During outdoor measurements, additional environmental variables were recorded, including dew point (°C), light intensity (lux), ultraviolet index (UVI), wind speed (m/s), wind direction (°), atmospheric pressure (mmHg), and rainfall (mL). The monitoring station was positioned adjacent to the exercise arena throughout the study.

### Data processing

Environmental data were downloaded from cloud storage in Microsoft Excel 2021 (Microsoft Corporation, Redmond, WA, USA) format. Environmental variables and timestamps were synchronized with the corresponding temperature measurements. Timestamps were rounded to the nearest 15-min interval using the MROUND function in Microsoft Excel, after which the environmental and temperature datasets were merged for statistical analysis.

All thermometers and the TC were calibrated according to the manufacturers' instructions before the study. All temperature measurements were recorded in degrees Celsius (°C). 

### Statistical analysis

Statistical analyses were performed using R version 4.5.2 (R Foundation for Statistical Computing, Vienna, Austria) and RStudio Build 418 (Posit Software PBC, Boston, MA, USA). Descriptive and inferential statistical analyses were conducted using the lme4 package [32].

Temperature measurements were analyzed using linear mixed-effects models. The initial maximal model included measurement method, time point, exercise activity, and body side as fixed effects, together with the interaction between measurement method and time point. Horse identification was included as a random effect to account for repeated measurements obtained from individual animals. Residual and quantile-quantile (Q-Q) plots were visually inspected to verify assumptions of homoscedasticity and normality.

Model simplification was performed using a backward stepwise approach based on likelihood ratio tests implemented in the lmerTest package [33], thereby minimizing overfitting while retaining biologically meaningful variables. Measurement method and anatomical region were combined into a single factor. The final model included measurement method, time point, and their interaction as fixed effects. The complete model output is provided in Supplementary Table S1.

Analysis of variance (ANOVA) followed by Bonferroni-adjusted pairwise comparisons was performed to determine statistical significance among fixed effects. Significant interactions were further explored using the emmeans package [34] to evaluate the effect of measurement method within each activity level and the effect of activity within each measurement method.

RT data were analyzed separately because outdoor RT measurements were unavailable for safety reasons. Statistical power was evaluated using 1,000 Monte Carlo simulations implemented in the simr package [35], demonstrating a statistical power of at least 99.63% for detecting a 1°C temperature difference.

Agreement among measurement methods was evaluated using Bland-Altman analysis, with RT serving as the reference standard whenever available. Within-subject standard deviation (WSD) was calculated from repeated measurements obtained from the same horse and was subsequently used to determine the repeatability coefficient (RC). Proportional bias was assessed through Bland-Altman plot inspection and simple linear regression of the measurement differences against the corresponding reference values. Relationships between measurement methods and environmental variables were evaluated using Kendall's rank correlation coefficient because of its suitability for data containing tied observations. A value of p ≤ 0.05 was considered statistically significant.

## RESULTS

### Environmental conditions

Each record included horse signalment, exercise activity, temperature measurements, and corresponding environmental conditions. Temperature measurements comprised values obtained using the TC from six anatomical regions (eye, neck, shoulder, back, trunk, and hindquarters), the IR at the medial canthus of the eye, and RT. A summary of the environmental conditions recorded during the study is presented in Table 2.

**Table 2:** Environmental conditions recorded during the study.

**Environmental parameter**	**Indoor**	**Outdoor**
Ambient temperature (°C)	24.44 ± 3.13	33.26 ± 2.03
Relative humidity (%)	64.26 ± 5.36	61.17 ± 7.99
Dew point (°C)	—	24.62 ± 0.87
Light intensity (lux)	—	27,043.79 ± 31,677.63
Ultraviolet index	—	1.65 ± 2.32
Wind speed (m/s)	—	0.66 ± 0.56
Wind direction (°)	—	112.25 ± 46.61
Atmospheric pressure (mmHg)	—	756.45 ± 1.68
Rainfall (mL)	—	0

Data are presented as mean ± SD.

### Temperature distribution

Neither exercise activity nor body side significantly influenced temperature measurements. In contrast, measurement method (\({η}_{p}^{2}\) = 0.05), sampling time point (\({η}_{p}^{2}\) = 0.51), and the interaction between measurement method and time point (\({η}_{p}^{2}\) = 0.13) significantly affected temperature measurements (all p ≤ 0.001).

Temperature values obtained using the three measurement methods differed significantly (Table 3). During the indoor examinations, RT consistently produced the highest temperatures, followed by IR measurements, whereas TC measurements obtained from the medial canthus of the eye yielded the lowest temperatures (all p ≤ 0.001).

**Table 3:** Temperatures measured using RT, IR, and TC at the medial canthus during the pre- and post-examination room assessments in 31 horses (n = 31).

**Temperature type**	**Pre-examination room**	**Post-examination room**
RT	37.53 ± 0.43ᵃ	37.86 ± 0.53ᵃ
IR	36.51 ± 0.28ᵇ	36.39 ± 0.29ᵇ
TC	34.65 ± 1.11ᶜ	35.00 ± 1.48ᶜ

Values are presented as mean ± standard deviation. RT = Rectal temperature; IR = Infrared thermography; TC = Thermal camera. Different superscript letters (ᵃ–ᶜ) within the same column indicate significant differences among temperature measurement methods (p ≤ 0.001).

Under indoor resting conditions, surface temperatures measured by TC were relatively uniform across the body, with the eye consistently exhibiting the highest temperature among all anatomical regions. Outdoor measurements obtained before exercise were significantly higher than those recorded indoors, and TC values exceeded IR measurements at all corresponding body regions (Table 4).

Following exercise, surface temperatures increased significantly in all TC-measured regions (all p ≤ 0.001). The greatest increases were observed in the back, trunk, and hindquarters, demonstrating marked regional heat accumulation after exercise. These findings highlight the ability of TC to visualize regional heat distribution following physical activity. One hour after exercise, surface temperatures had largely returned to pre-exercise values; however, the shoulder and trunk retained relatively higher temperatures than the remaining body regions.

**Table 4:** Temperatures measured using IR at the medial canthus and TC at different anatomical regions before and after exercise in 31 horses (n = 31).

**Temperature type**	**Pre-examination room (Indoor)**	**Pre-exercise (Outdoor)**	**Post-exercise (Outdoor)**	**Post-examination room (Indoor)**
IR	36.51 ± 0.28ᵃˣ	36.58 ± 0.22ᵃˣ	36.94 ± 0.57ᵇˣ	36.39 ± 0.29ᵃˣ
TC				
Eye	34.65 ± 1.10ᵇʸ	37.40 ± 1.77ᵃˣ	37.91 ± 1.52ᵃˣ	35.00 ± 1.48ᵇʸ
Neck	33.73 ± 1.70ᶜʸ	37.06 ± 1.93ᵃˣ	37.69 ± 1.64ᵃᵇˣ	34.03 ± 2.19ᶜʸ
Shoulder	33.73 ± 1.70ᶜʸ	37.15 ± 2.11ᵃˣ	37.81 ± 1.70ᵃˣ	34.23 ± 2.16ᵇᶜʸ
Back	33.38 ± 1.89ᶜᶻ	37.01 ± 1.96ᵃʸ	37.93 ± 1.60ᵃˣ	33.83 ± 2.53ᶜᶻ
Trunk	33.78 ± 1.69ᶜᶻ	37.39 ± 2.25ᵃʸ	38.21 ± 1.57ᵃˣ	34.34 ± 2.16ᵇᶜᶻ
Hindquarters	33.33 ± 1.80ᶜᶻ	37.22 ± 2.09ᵃʸ	38.17 ± 1.51ᵃˣ	33.94 ± 2.29ᶜᶻ

Values are presented as mean ± standard deviation. IR = Infrared thermography; TC = Thermal camera. Within the same row, different superscript letters (ᵃ–ᶜ) indicate significant differences among measurement periods/conditions (p ≤ 0.001). Within the same column, different superscript letters (ˣ–ᶻ) indicate significant differences among temperature measurement sites/methods (p ≤ 0.001).

### Method agreement

Agreement among the different temperature measurement methods was evaluated using Bland-Altman analysis. Data collected during the pre-examination room assessment were used because they represented baseline physiological conditions. The Bland-Altman plots illustrating the agreement between RT and each temperature measurement method are presented in Figure 6.

Compared with RT, IR measurements obtained at the medial canthus underestimated temperature by a mean of 1.02°C, with a WSD of 0.77°C and an RC of 2.13°C (95% limits of agreement [LOA]: −1.75°C to −0.30°C) (Table 5). In contrast, TC measurements obtained from the eye demonstrated a larger negative bias relative to RT, with a mean difference of 2.88°C, a WSD of 2.19°C, and an RC of 6.08°C (95% LOA: −5.13°C to −0.63°C). The remaining anatomical regions measured using TC exhibited even larger differences from RT, indicating lower agreement than the eye region.

Proportional bias was detected for both the IR-RT comparison (β = −0.67; 95% confidence interval [CI]: −0.82 to −0.52; p ≤ 0.001) and the TC eye-RT comparison (β = −0.80; 95% CI: −1.47 to −0.13; p ≤ 0.05).

Direct comparison between TC and IR also demonstrated lower temperatures for TC at the eye region, with a mean difference of 1.86°C, a WSD of 1.50°C, and an RC of 4.17°C (95% LOA: −3.89°C to 0.18°C) (Table 6). Similar trends were observed for all remaining anatomical regions.

Correlation analysis demonstrated a moderate positive relationship between RT and IR measurements (r = 0.40; 95% CI: 0.26–0.54; p ≤ 0.001). However, no significant correlation was observed between RT and TC measurements or between IR and TC measurements. Relative to RT, the estimated measurement error was 2.81 ± 1.02% for IR and 8.42 ± 3.63% for TC.

**Figure 6:**
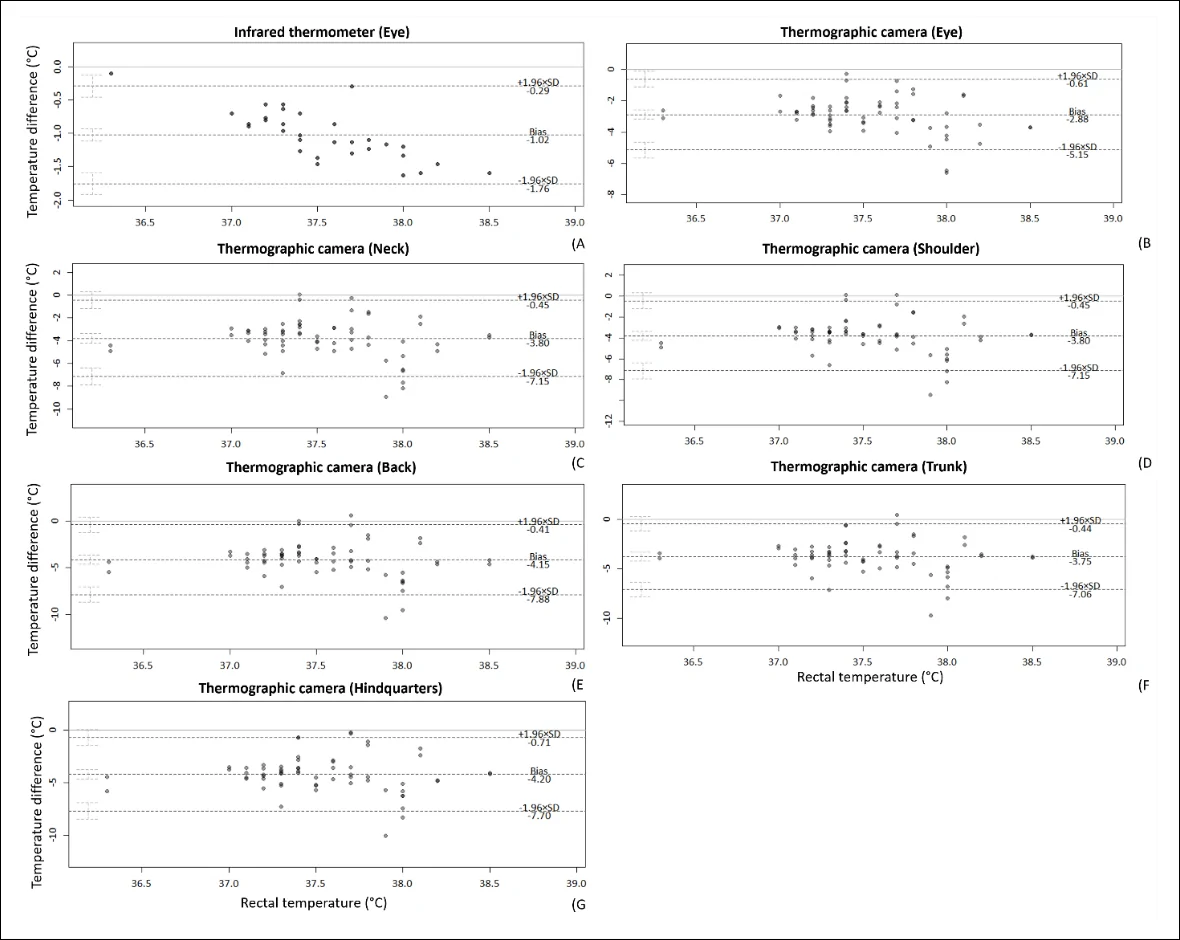
Bland-Altman plots showing agreement between RT and (A) IR measurements at the medial canthus and TC measurements at the (B) eye, (C) neck, (D) shoulder, (E) back, (F) trunk, and (G) hindquarters in 31 horses (n = 31). The y-axis represents the difference between RT and IR or TC measurements (°C), whereas the x-axis represents RT (°C). The solid gray line indicates the mean difference, and the dashed lines indicate the 95% LOA (±1.96 SD). RT = Rectal temperature. IR = Infrared thermometer. TC = Thermographic camera. LOA = Limits of agreement. SD = Standard deviation.

**Table 5:** Bland-Altman analysis comparing IR and TC measurements with RT during the pre-examination room assessment in 31 horses (n = 31).

Temperature type	Mean temperature (°C)	Mean difference (°C)	SD of difference	Lower LOA (°C)	Upper LOA (°C)	WSD (°C)	RC (°C)
RT	37.53	—	—	—	—	—	—
IR	36.51	−1.02	0.37	−1.75	−0.30	0.77	2.13
**TC**							
Eye	34.65	−2.88	1.15	−5.13	−0.63	2.19	6.08
Trunk	33.78	−3.75	1.68	−7.04	−0.47	2.91	8.05
Back	33.38	−4.15	1.89	−7.85	−0.44	3.22	8.93
Shoulder	33.73	−3.80	1.70	−7.12	−0.48	2.94	8.16
Neck	33.73	−3.80	1.70	−7.13	−0.47	2.94	8.16
Hindquarters	33.33	−4.20	1.77	−7.67	−0.74	3.23	8.94

Data are presented as mean or mean difference. LOA = Limits of agreement. WSD = Within-subject standard deviation. RC = Repeatability coefficient. RT = Rectal temperature. IR = Infrared thermometer. TC = Thermographic camera.

**Table 6:** Bland-Altman analysis comparing TC measurements with IR during the pre-examination room assessment in 31 horses (n = 31).

Temperature type	Mean temperature (°C)	Mean difference (°C)	SD of difference	Lower LOA (°C)	Upper LOA (°C)	WSD (°C)	RC (°C)
IR	36.51	—	—	—	—	—	—
**TC**							
Eye	34.65	−1.86	1.04	−3.89	0.18	1.50	4.17
Trunk	33.78	−2.73	1.59	−5.85	0.39	2.23	6.19
Back	33.38	−3.12	1.81	−6.66	0.41	2.55	7.07
Shoulder	33.73	−2.78	1.63	−5.96	0.41	2.28	6.31
Neck	33.73	−2.78	1.63	−5.98	0.42	2.28	6.31
Hindquarters	33.33	−3.18	1.70	−6.51	0.15	2.55	7.07

Data are presented as mean or mean difference. LOA = Limits of agreement. WSD = Within-subject standard deviation. RC = Repeatability coefficient. IR = Infrared thermometer. TC = Thermographic camera.

### Environmental effects

The influence of environmental conditions on temperature measurements was evaluated by examining the relationships between ambient temperature, relative humidity, and the differences among RT, IR, and TC measurements. Temperature differences were calculated by subtracting RT from both IR and TC measurements to determine the extent to which environmental factors affected each method.

No significant correlation was observed between ambient temperature or relative humidity and the difference between IR and RT measurements, indicating that IR measurements were relatively unaffected by environmental conditions within the range encountered during this study. In contrast, a weak positive correlation was identified between ambient temperature and the difference between TC and RT measurements (r = 0.24; 95% CI: 0.12–0.36; p ≤ 0.001), whereas no meaningful association with relative humidity was detected.

Because IR measurements were not influenced by environmental conditions, they were subsequently used as the reference method for evaluating environmental effects on TC measurements across both indoor and outdoor assessments. Analysis of TC-IR temperature differences demonstrated a moderate positive correlation with ambient temperature (r = 0.52; 95% CI: 0.45–0.59; p ≤ 0.001) and a weak negative correlation with relative humidity (r = −0.13; 95% CI: −0.23 to −0.04; p ≤ 0.01). Similar environmental effects were observed across all anatomical regions evaluated using TC (Figure 7).

**Figure 7:**
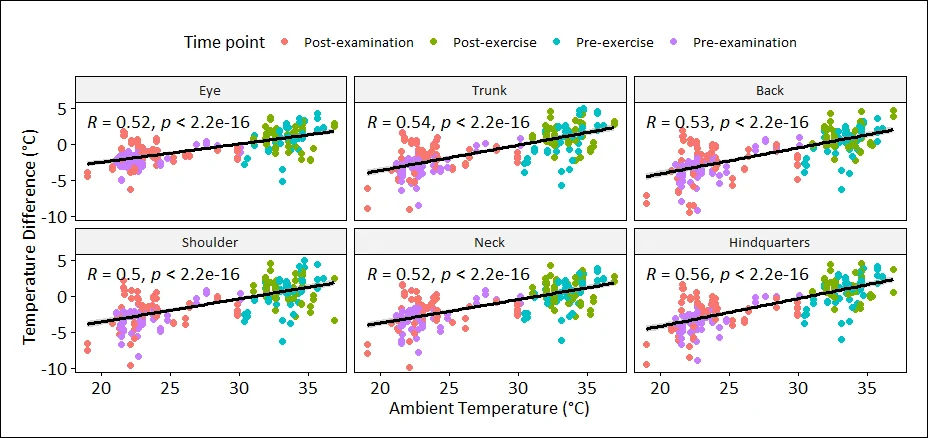
Correlations between ambient temperature and the differences between TC and IR measurements for each anatomical region. The y-axis represents the temperature difference between TC and IR (°C), whereas the x-axis represents ambient temperature (°C). Correlation coefficients and corresponding p-values are shown within each panel. TC = Thermographic camera. IR = Infrared thermometer.

## DISCUSSION

To our knowledge, this is among the first studies to systematically compare TC, IR, and RT in exercising horses under practical tropical climatic conditions. The mean RT recorded before and after exercise (37.53 ± 0.43°C and 37.86 ± 0.53°C, respectively) was consistent with the normal physiological range previously reported for healthy horses (37.1 ± 0.51°C) [36]. Likewise, the mean IR and TC eye temperatures were comparable to those reported in previous equine studies [24, 37]. In contrast, TC measurements obtained from the neck, shoulder, back, and hindquarters were substantially higher than those reported under temperate environmental conditions [13]. These findings are likely attributable to the considerably higher ambient temperatures encountered in tropical climates, demonstrating that environmental conditions markedly influence surface temperature measurements obtained using thermography.

### Comparison of temperature measurement methods

Among the three temperature measurement methods evaluated, RT consistently produced the highest temperature values because it reflects core body temperature, which is tightly regulated by hypothalamic thermoregulatory mechanisms and represents the thermal status of internal organs [1]. In contrast, IR and TC measure skin surface temperature, which is influenced by local tissue metabolism, peripheral blood flow, and environmental conditions [11]. The medial canthus exhibited the highest surface temperature among all anatomical regions, consistent with previous reports in horses, cattle, and pigs [22, 23, 38, 39]. This region is relatively hairless and highly vascularized, allowing its surface temperature to closely reflect changes in blood perfusion associated with core thermoregulation.

Although the medial canthus showed the closest agreement with RT, proportional bias was identified between IR and RT measurements, indicating that the degree of underestimation increased as RT increased. This finding suggests that eye temperature reflects only part of the physiological changes occurring in core body temperature, particularly at higher temperatures. Furthermore, symmetrical temperature distributions between the left and right sides of the body and among corresponding anatomical regions were observed, corroborating previous thermographic studies in horses [14, 22].

### Effects of exercise on surface temperature

Exercise produced a marked increase in TC-measured surface temperatures, particularly over the back, trunk, and hindquarters. These regions contain large muscle groups that generate substantial metabolic heat during locomotion, explaining the greater temperature elevations observed after exercise. Unlike IR, which provides a single-point measurement, TC enabled visualization of regional heat distribution across the body, demonstrating its ability to identify localized thermal changes associated with muscular activity.

One hour after exercise, temperatures measured using TC had largely returned to baseline values, although slightly elevated temperatures persisted over the shoulder and trunk. This recovery pattern agrees with previous findings in exercising horses [25] and indicates that healthy horses are capable of dissipating exercise-induced heat efficiently, even under tropical environmental conditions. These baseline recovery data may be clinically valuable for identifying horses with impaired heat dissipation resulting from inadequate cooling, musculoskeletal injury, inflammation, or other pathological conditions that delay the return to normal surface temperature.

### Environmental influences on temperature measurements

The present study demonstrated that environmental conditions affected TC measurements considerably more than IR measurements. IR showed good agreement with RT supporting previous reports that IR can provides a estimate of RT temperature [24]. We also found that IR was not significantly influenced by ambient temperature or humidity, IR provides consistence temperature estimation under a wide range of environmental conditions. Conversely, TC measurements were significantly associated with ambient temperature, confirming previous observations that thermographic imaging is highly susceptible to environmental influences [22, 27, 28]. More importantly, this study quantified the magnitude of these effects under tropical climatic conditions.

The results showed that TC underestimated surface temperature under relatively lower ambient temperatures, whereas increasing ambient temperature progressively reduced this difference until TC measurements eventually exceeded those obtained using IR. This phenomenon can be explained by the operating principle of thermographic cameras. TC detects infrared radiation emitted from both the target object and the surrounding environment. Consequently, changes in background radiation directly influence the detected signal. Lower ambient temperatures reduce background infrared radiation, thereby contributing to lower measured temperatures, whereas higher ambient temperatures increase the total infrared radiation received by the detector, resulting in higher temperature estimates [40].

A weak negative correlation between relative humidity and TC measurements was also observed. Increased atmospheric water vapor can absorb part of the emitted infrared radiation before it reaches the detector, thereby reducing the measured surface temperature [40]. Although this effect was smaller than that of ambient temperature, it should nevertheless be considered when interpreting thermographic images obtained under humid tropical conditions.

Another factor contributing to the superior performance of IR may be the shorter measurement distance. In the present study, IR measurements were obtained at approximately 0.5 cm from the eye, whereas TC images were acquired at a standardized distance of 0.5 m. Previous studies have shown that increasing measurement distance reduces the proportion of infrared radiation originating directly from the target while increasing the influence of background radiation [40]. The focal point measurement used by IR therefore minimizes environmental interference and likely explains its closer agreement with RT compared with TC. Despite using relatively affordable and compact instruments, the present study produced findings comparable with those reported using more sophisticated equipment, demonstrating the growing accessibility and practical applicability of these technologies for equine clinical practice.

### Study limitations

Several limitations should be considered when interpreting the present findings. First, exercise activities were not completely standardized because horses followed their routine riding schedules, resulting in variation in exercise type and duration. Although this reflects practical field conditions, it may have contributed to variability in temperature measurements. Second, all horses had short hair coats with limited coat color variation, preventing evaluation of the influence of different coat characteristics on thermographic measurements. Third, RT was not measured immediately before or after exercise because of safety concerns, precluding direct comparison between RT and TC under outdoor conditions. Fourth, saddle placement during exercise prevented thermographic assessment of the cranial dorsal region immediately before and after exercise. Finally, data were collected during a single season; therefore, the effects of broader seasonal and climatic variation could not be evaluated. Future studies incorporating standardized exercise protocols, greater diversity in coat characteristics, repeated seasonal measurements, and safe methods for assessing core temperature during exercise would further strengthen the clinical application of thermographic imaging in horses.

## CONCLUSION

This study demonstrated that RT remained the highest and most stable indicator of core body temperature in clinically healthy horses, whereas IR provided the closest approximation to RT and showed substantially better agreement than TC. Although TC consistently underestimated RT under indoor conditions, it effectively characterized regional surface temperature distribution and detected exercise-induced thermal changes, particularly over the back, trunk, and hindquarters. Surface temperatures increased significantly immediately after exercise and returned close to baseline within 1 h, indicating efficient thermoregulation in healthy horses under tropical climatic conditions. Furthermore, ambient temperature significantly influenced TC measurements, whereas IR measurements remained relatively unaffected by environmental conditions, highlighting the greater robustness of IR for estimating body temperature under field conditions.

The findings have important practical implications for equine clinical practice and sports medicine. IR offers a rapid, reliable, and environmentally resilient method for routine non-contact temperature assessment when estimation of core body temperature is required. In contrast, TC provides unique advantages by enabling visualization of regional heat distribution that cannot be detected using point measurements alone, making it particularly valuable for monitoring exercise responses, identifying abnormal heat accumulation, evaluating recovery following exercise, and supporting the early detection of musculoskeletal injury or inflammation. However, environmental conditions, particularly ambient temperature, should be carefully considered when interpreting thermographic images obtained under tropical conditions.

A major strength of this study is that it was conducted under practical riding club conditions in a tropical climate, thereby providing clinically relevant evidence for the application of non-contact temperature measurement techniques outside controlled laboratory environments. The repeated-measures within-subject design minimized inter-individual variation, while simultaneous evaluation of RT, IR, and TC allowed comprehensive comparison of measurement accuracy, agreement, and environmental influences. In addition, this study quantified the magnitude of environmental effects on thermographic measurements, providing valuable information for interpreting TC findings in tropical regions.

Several limitations should be acknowledged. Exercise intensity and duration were not completely standardized because horses followed their routine riding activities. The study population consisted primarily of short-coated horses with limited coat color variation, restricting evaluation of coat characteristics on thermal measurements. RT could not be measured immediately before or after exercise because of safety considerations, preventing direct comparison between RT and TC under outdoor conditions. In addition, saddle placement limited thermographic assessment of portions of the dorsal back, and the study was conducted during a single season, which may not fully represent the range of environmental conditions encountered throughout the year.

Future studies should investigate these measurement techniques across different seasons, climatic conditions, breeds, coat lengths, and coat colors while incorporating standardized exercise protocols and larger study populations. Further research should also evaluate the diagnostic performance of TC and IR in horses with musculoskeletal disorders, inflammatory diseases, and heat-related illnesses and explore calibration or environmental correction models to improve the accuracy of thermographic measurements under varying environmental conditions.

Overall, IR is the most suitable non-contact method for estimating body temperature under tropical field conditions because of its close agreement with RT and minimal environmental influence. Although TC is less suitable as a surrogate for core body temperature, its ability to visualize regional heat distribution provides valuable complementary information for exercise monitoring and clinical assessment. When interpreted with appropriate consideration of environmental conditions, TC represents a practical adjunctive imaging tool for equine health evaluation, welfare monitoring, and sports medicine in tropical environments.

## DATA AVAILABILITY

The supplementary data can be made available from the correspond author upon request.

## GENERATIVE AI DECLARATION

The authors declare that generative artificial intelligence (AI) tools were used solely to improve language, grammar, and readability during manuscript preparation. All scientific content, data analysis, interpretation of results, and conclusions were developed and verified by the authors. The authors take full responsibility for the accuracy, integrity, and originality of the work presented, and no AI tool was listed as an author. Generative AI was used to create horse picture in figure 3.

## AUTHORS’ CONTRIBUTIONS

JP: Conceptualization, methodology, data collection, data curation, formal data analysis, and writing—original draft preparation. AI: Methodology, data collection, and writing—review and editing. PS and KW: Data collection. NS: Methodology. WP and PR: Methodology, supervision, and writing—review and editing. KNL: Methodology, formal data analysis, validation, and writing—review and editing. All authors have read and approved the final version of the manuscript.
